# Detection of Environmentally Harmful Malathion Pesticides Using a Bimetallic Oxide of CuO Nanoparticles Dispersed over a 3D ZnO Nanoflower

**DOI:** 10.3390/ma16227065

**Published:** 2023-11-07

**Authors:** Lakshmanan Gurusamy, Ru-Wen Cheng, Sambandam Anandan, Cheng-Hua Liu, Jerry J. Wu

**Affiliations:** 1 Department of Environmental Engineering and Science, Feng Chia University, Taichung 407, Taiwan; guru.samy665@gmail.com (L.G.); rita19950303@gmail.com (R.-W.C.); chhengliu@fcu.edu.tw (C.-H.L.); 2 Department of Chemistry, National Institute of Technology, Trichy 620015, India; sanand99@yahoo.com

**Keywords:** nonenzymatic, electrochemical sensor, malathion, 3D nanoflower, pesticides

## Abstract

Super-sensitive malathion detection was achieved using a nonenzymatic electrochemical sensor based on a CuO/ZnO-modified glassy carbon electrode (GCE). Due to the high affinity between the Cu element and the sulfur groups in malathion, the developed CuO-ZnO/GCE sensor may bond malathion with ease, inhibiting the redox signal of the Cu element when malathion is present. In addition to significantly increasing the ability of electron transfer, the addition of 3D-flower-like ZnO enhances active sites of the sensor interface for the high affinity of malathion, giving the CuO-ZnO/GCE composite an exceptional level of sensitivity and selectivity. This enzyme-free CuO-ZnO/GCE malathion sensor demonstrates outstanding stability and excellent detection performance under optimal operating conditions with a wide linear range of malathion from 0 to 200 nM and a low detection limit of 1.367 nM. A promising alternative technique for organophosphorus pesticide (OP) determination is offered by the analytical performance of the proposed sensor, and this method can be quickly and sensitively applied to samples that have been contaminated with these pesticides.

## 1. Introduction

The extreme toxicity of pesticides employed in modern agriculture poses a potential threat to our health, the environment, and food safety [1,2,3]. Although organophosphorus pesticides, such as methyl parathion, dimethoate, and malathion, have been frequently used for their effectiveness in agricultural production, they may cause environmental pollution due to excess pesticide residue problems. A typical pesticide containing organophosphorus is malathion, which is also extensively utilized in agriculture due to its simple disintegration, lack of buildup in animals and plants, low environmental impact, and excellent insecticidal effectiveness. Traditional methods for pesticide detection are based on gas chromatography (GC), high-performance liquid chromatography (HPLC), enzyme-linked immunosorbent assays, etc. [4,5,6]. Despite the established procedures, major drawbacks of these include the cost and the testing time with operational difficulties. In recent years, to meet the need for rapid and accurate analysis of targets, electrochemical sensors have been substantially developed that possess the advantages of high sensitivity, high selectivity, and low cost [7,8]. Enzymatic electrochemical biosensors, in particular, are widely employed because of their great selectivity, sensitivity, and specificity. However, due to the poor stability and sensitivity of enzymes to environmental changes, such as acidity, temperature, and storage conditions, their applications are still rather limited. Enzyme deactivation and sensor malfunction can be caused by harsh environmental conditions. Therefore, it is crucial to design nonenzymatic electrochemical sensors for malathion detection that are more stable and require a simpler preparation technique [9,10,11].

The high sensitivity and low detection limits of nonenzymatic electrochemical sensors were developed through a variety of new nanomaterials with various physiochemical properties, such as MXene-based nanocomposites, perovskites, noble metal materials, metal–organic frameworks (MOFs), and pyrochlore oxide. Among these, the metal oxides-based electrode materials are environmentally friendly, low-cost, and easy to prepare. Nonenzymatic electrochemical sensors such as various metal oxides, for instance, CuO, ZnO, Fe_2_O_3_, Co_3_O_4_, and NiO, have been employed because of their excellent electrocatalytic activity, great abundance, low cost, and their ability to conduct electrochemical reactions at lower potentials [12,13]. The p-type semiconducting material of copper oxide (CuO) has been used in different applications in supercapacitors, electrochemical sensors, CO_2_ reduction, and photocatalysts due to its inexpensiveness, ease of storage, and high specific capacitance properties [14,15,16]. CuO-based nanomaterials have been used in electrochemical sensors to detect various organic and inorganic molecules and pollutants, for instance, the application of CuO nanoparticles onto multi-walled carbon arrays as glucose sensors [17,18]. Initially, a hybrid nanocomposite including single-walled carbon nanotubes (SWCNTs) and copper-oxide nanowires (CuO NWs) was used to create a novel electrochemical sensor for the detection of organophosphorus pesticides (OPs) [19,20].

The ability of transition metals and metal oxides to facilitate electron transfer processes at low potential, as well as their exceptional electrocatalytic activity, high stability, and affordability, have recently attracted more interest as electrode system modifiers [21,22]. The creation and production of 3D-flower-like ZnO nanostructures have grown in significance in recent years because of their large surface area and favorable electrical characteristics. By pushing past the constraints imposed by their distinctive atom and architectural arrangements, 3D-flower-like ZnO nanostructures at the micro/nanoscale open new doors to electrochemical sensing applications. When compared to pristine ZnO and CuO nanoparticles, combining CuO and ZnO nanoparticles is likely to result in an effective nanocomposite that drives the sensing response and lengthens the lifetime of the charge carriers [23,24,25,26]. Due to their morphological, optical, and electrical properties, ZnO–CuO nanocomposites are more useful in key scientific applications than undoped nanomaterials. Because of its strong electrocatalytic activity, large specific surface area, low toxicity, and great sensitivity to malathion, CuO is a desirable material. Due to numerous donors of hetero atoms (S, O, and P), transition metal ions can donate electrons with malathion to produce a soluble metal–malathion complex. This feature can be used for the quantitative determination of malathion. Due to its high specific surface area, low cost, low toxicity, and electrocatalytic activity, copper (II) oxide (CuO) stands out as an appealing alternative material to increase the sensitivity of electrochemical detection of malathion through the development of a soluble metal–malathion complex on a copper electrode and the use of DPV to show an oxidative current for malathion. Therefore, CuO nanoparticles are a material that holds promise for usage in electrode modification for malathion detection by increasing the rate of electron transfer and catalytic activity of the modified electrode for malathion [27,28,29,30].

**Scheme 1:**
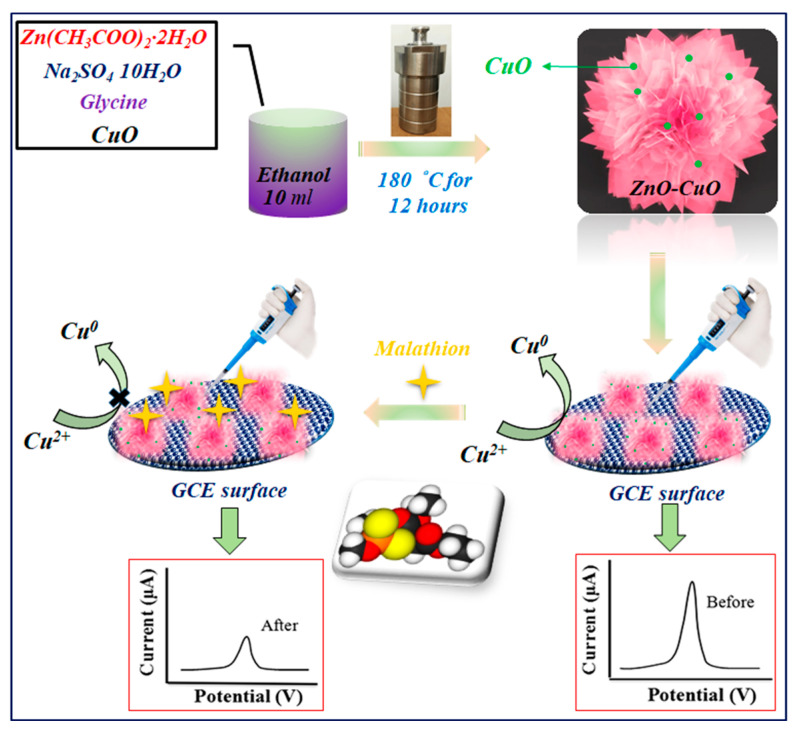
Schematic representation of the synthesis and construction of ZnO–CuO/GCE for the nonenzymatic electrochemical detection of malathion.

In this paper, we report that a bimetallic oxide of CuO nanoparticles dispersed on a 3D nanoflower of ZnO was prepared via the hydrothermal method, as seen in Scheme 1, which depicts the electrochemical characterization of the as-prepared ZnO–CuO modified electrode and its application for the determination of malathion. Then, ZnO in various weight proportions ranging from 0 to 80% was added to the CuO nanoparticles that were created for the optimization investigation. The exceptional catalytic properties of the as-prepared ZnO–CuO nanocomposite allowed for the highly sensitive quantitative detection of malathion over a wide linear range from 0 to 200 nM and a low detection limit of 1.367 nM. The proposed sensor is commercially viable for real-time applications because of a straightforward synthetic process and the utilization of inexpensive materials.

## 2. Materials and Methods

Copper acetate monohydrate (Cu(OAc)_2_) (99%), Zn(CH_3_COO)_2_·2H_2_O (98%), disodium hydrogen phosphate (Na_2_HPO_4_), glycine, Na_2_SO_4_·10H_2_O (≥99.5%), and malathion (99%) were obtained from Sigma Aldrich (St. Louis, MI, USA). Anhydrous ethanol (EtOH, ≥98.7%) and NaOH (98%) were purchased from Merck Ltd. (Taipei, Taiwan). 0.1 M Na_2_HPO_4_-citric acid buffer solution was made by mixing 0.2 M Na_2_HPO_4_ and 0.1 M citric acid in a certain proportion. Malathion solution was prepared with methanol, and other solutions were prepared with double distilled water.

The electrochemical measurements were conducted in a three-electrode system, which was carried out by an Autolab PGSTAT128N electrochemical workstation (Metrohm Inc., Herisau, Switzerland). The working electrode was a bare or modified glassy carbon electrode (GCE). A saturated calomel electrode worked as the reference electrode, while a platinum wire served as the auxiliary electrode. The feature of FE–SEM (JEOL JSM-7800F, Saga, Japan) has higher magnification, larger depth of focus, and greater resolution that reveals details about 1.0 nm (15 kV) and 2.2 nm (1 kV) in size and offers easier observation of three-dimensional micro-structure images for sample surface and specific surface area determined by the Brunauer–Emmett–Teller (BET) nitrogen adsorption–desorption (Nova 2000E, Anton Paar Gmbh, Graz, Austria). X-ray diffraction (XRD) patterns were obtained in an X-ray diffractometer (Applied Rigaku Technologies Inc., Cedar Park, TX, USA) using CuKa irradiation (1.5406 Å) at a scanning rate of 20 min. The accelerating voltage and the applied current were 40 kV and 80 mA, respectively. XPS analysis measurement (Shimadzu, Tokyo, Japan) was performed using Kratos Axis Ultra DLD X-ray photoelectron spectroscopy (Texas Materials Institute, Austin, TX, USA) equipped with a 180° hemispherical sector analyzer with a 165 mm mean radius. X-rays were produced by an Al monochromator.

The catalyst under pure zinc oxide and copper oxide, if compared with the composite catalyst, will improve the ability of organophosphorus pesticides in water. Thus, the following two composite catalysts were synthesized. (a) ZnO/CuO: 0.439 g of Zn (CH_3_COO)_2_·2H_2_O (0.04 M), 0.4 g of glycine, and 0.4 g of Na_2_SO_4_·10H_2_O were completely dissolved in 15 mL of deionized water and 10 mL of ethanol while vigorously stirring for 5 min. Then, 0.4 g of NaOH was added to the prepared solution to reach pH = 10 under magnetic stirring for about 1 h. Next, copper oxide was added in the required proportion, and the mixed solution was poured into a 45 mL Teflon-lined stainless-steel autoclave and heated at 180 °C for 12 h. After the autoclave was naturally cooled to room temperature, the precipitate was collected by centrifugation, washed several times with distilled water and absolute ethanol, dried in air at 80 °C for 12 h, and calcined at 400 °C for 2 h. For a comparison study, the different weight % of ZnO at a fixed concentration of CuO was prepared. (b) CuO/ZnO: 0.54 g of copper acetate (0.06 M) was stirred in 80 °C distilled water for 2 min to ensure complete dissolution. The required proportion of zinc oxide was added, and then 30 mL of 2 M NaOH (pH = 10) was dropped into the above solution through a dropper to produce a black precipitate. After stirring for 20 min, we removed the composites and let them cool, then filtered and dried them.

The uniform suspension was prepared by 2 mg of as-prepared electrode materials (ZnO–CuO) dispersed in 1 mL of deionized water and then added 100 µL of Nafion under ultrasonic irradiation. Before modifying the electrode, we first polished the bare GCE with an alumina slurry to obtain a mirror-like surface, then covered the electrode surface with distilled water and alcohol, and sonicated for ten minutes, then cleaned the electrode several times with ethanol and distilled water to completely remove impurities. After drying, the 6 µL of ZnO–CuO suspension was modified with GCE using the drop-casting method and then dried at 70 °C.

Electrochemical experiments were measured in 5 mL of 0.1 M Na_2_HPO_4_-citric acid buffer solution (pH = 3.0). At a scan rate of 100 mV s^−1^, cyclic voltammetry was recorded at a potential range from −1 to +1 V. By scanning the potential range between −1 and +1 V, differential pulse voltammetry (DPV) was obtained. A frequency range of 0.1 to 10,000 Hz was used for electrochemical impedance spectra (EIS), and 5 mV of amplitude and 0.25 V of potential were present. According to the following formula, the relative current change (Δ*I*/*I*_0_) could be used to determine the inhibition ratio, which was the basis for the malathion detection.

(1) $$\frac{\Delta I}{I_{0}}=\left[\left(I_{0}-I)/I_{0}\right)\right]\times 100\;$$

where *I*_0_ and *I* are the anodic peak currents of the modified electrode contacted without and with malathion, respectively. The procedure for the preparation of ZnO–CuO/GCE and the electrochemical detection of malathion is illustrated in Scheme 1.

## 3. Results and Discussion

### 3.1. Characterization of Electrode Materials

**Figure 1:**
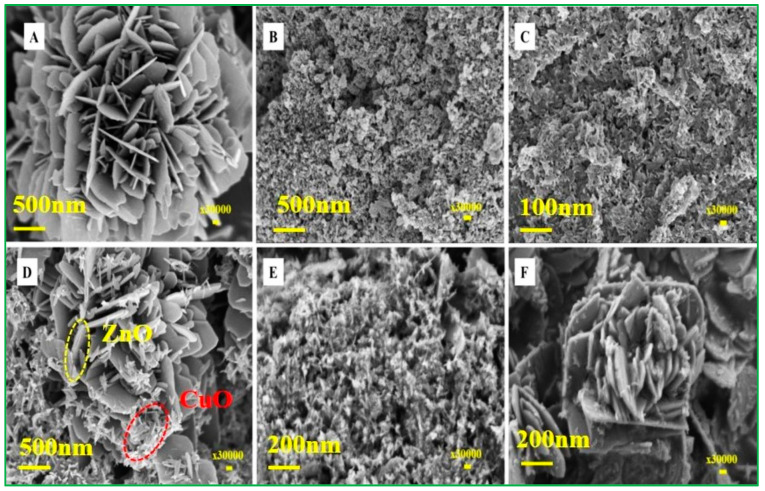
FE–SEM images of (**A**) ZnO/20% CuO, (**B**) CuO/10% ZnO, (**C**) CuO/20% ZnO, (**D**) CuO/40% ZnO, (**E**) CuO/60% ZnO, and (**F**) CuO/80% ZnO.

The morphology of metal-oxide composites for varied proportions of ZnO and CuO is shown in Figure 1. The SEM image of ZnO/20% CuO (Figure 1A) displays the exceptional morphology of the flower form of ZnO, but the smaller proportion of CuO is not shown in that SEM image. Meanwhile, the current value of the ZnO/20%CuO modified electrode utilized to detect malathion is slightly lower than that of the CuO/20% ZnO-modified electrode, which is compared in the CV data. As a result, raising the proportion of ZnO percentages from 10 to 80% (Figure 1B–F) resulted in a reasonably uniform and rough surface structure. As illustrated in Figure 1F, the flower-shaped zinc oxide was generated by multiple nanoplates aggregating at the nucleus center. The proportion of zinc oxide concentration increased as the thickness of the nanoplates increased, but there was a lower proportion of copper-oxide nanoparticles dispersed on the ZnO nanoplates.

**Figure 2:**
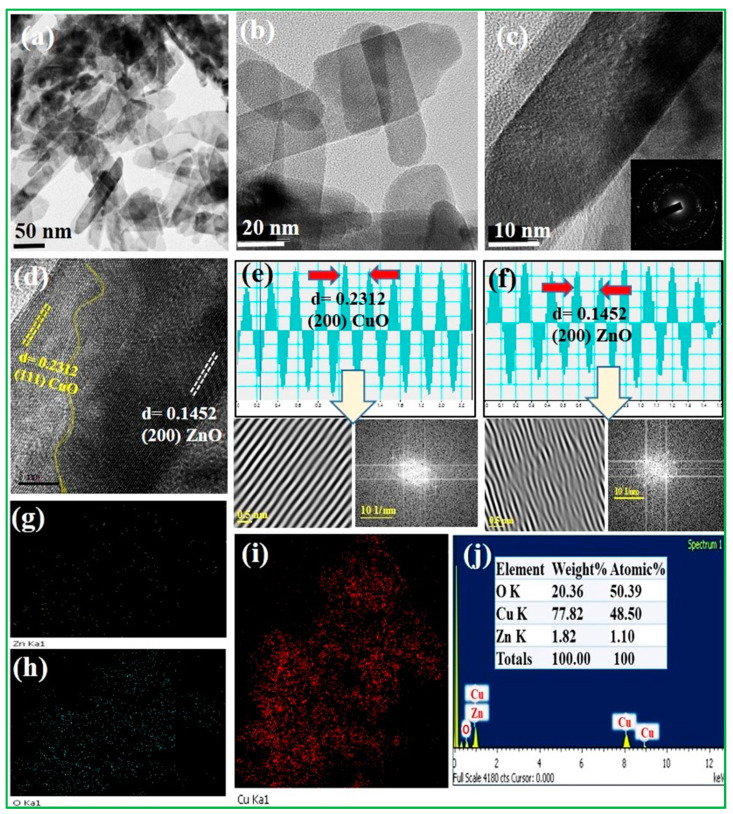
HR–TEM images of (**a**–**c**) CuO/20% ZnO for different magnification, (**d**–**f**) spacing values calculated by FFT and IFFT spectrum, and (**g**–**j**) EDX and elemental analysis of CuO/20% ZnO.

The morphologies of the CuO/20% ZnO electrocatalyst were investigated by HR–TEM (Figure 2). The 3D ZnO nanoflower was observed to have closely agglomerated nanoplates of varying sizes, as demonstrated by the TEM images (Figure 2a–c). The nucleation center of the 3D nanoflower of ZnO had not yet grown in CuO/20% ZnO nanocomposites due to the lower ZnO content. A 3D nanoflower of ZnO was produced when the percentage of ZnO was increased from 10% to 80% while CuO nanoparticles were present. The HR–TEM image (Figure 2c) reveals that the CuO/20% ZnO grains have spaces between them where various-sized nanoplates are densely clustered together. The selected area diffraction pattern (SAED) for CuO/20% ZnO material is polycrystalline in nature because of several bright spots dispersed over the ring pattern, as presented in inset Figure 2c. As shown in Figure 2d–f, the well-separated two different grains used to calculate the lattice fringes values through the FFT and IFFT spectrum of CuO/20% ZnO display interplanar spacings of 0.2312 and 0.1452 nm in the particle, which match separately those of the (111) and (200) planes of CuO and ZnO. The energy-dispersive X-ray spectroscopy (EDS) image shows that individual nanoparticles consist of Zn, Cu, and O uniformly distributed on the CuO/20% ZnO as revealed in Figure 2g–i. Furthermore, the EDX spectra (Figure 2j) confirm the presence of elements as well as atomic and weight percentages of Zn, Cu, and O in the CuO/20% ZnO. The pristine ZnO nanostructure and the CuO/20% ZnO nanostructure had BET surface areas of 41.509 m^2^g^−1^ and 92.864 m^2^g^−1^, respectively. Their mesoporous structure, which is composed of CuO and 20% ZnO, and their huge surface area suggest that they could be used in electrochemical sensors to improve the way that malathion pesticides are sensed.

**Figure 3:**
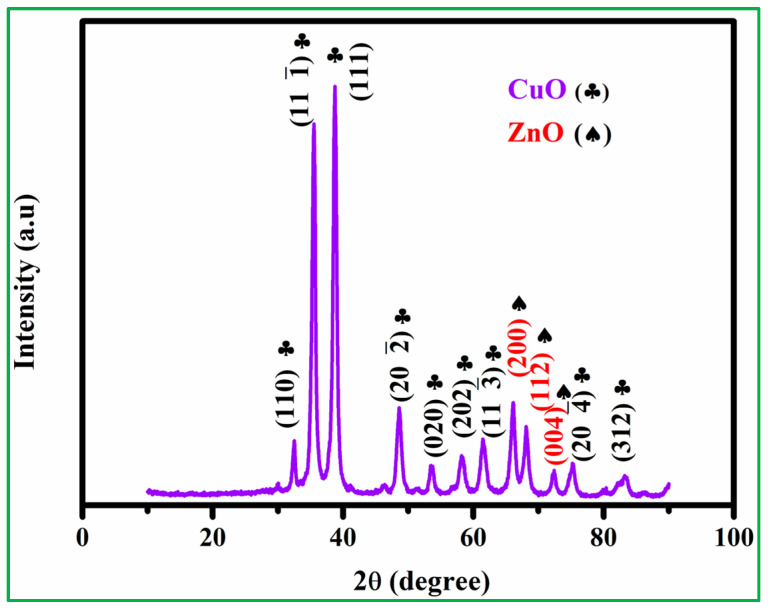
The XRD patterns of CuO/20% ZnO.

In the XRD crystal structure analysis, as shown in Figure S1A, it was found from the diffraction peak observation that the main diffraction 2θ positions are 31.76°,34.42°, 36.34°, 47.63°,56.62°, 62.83°, 66.28°, 67.92°, 72.58°, and 76.97°, which corresponds to JCPDS No. 36–1451, (100), (002), (101), (102), (110), (103), (200), (112), (201), (004), and (202) crystalline phase, where these peaks were determined to be polycrystalline properties of ZnO nanoparticles. Furthermore, the CuO crystal structure (Figure S1B) shows the different diffraction peaks observed at 32.3°, 35.4°, 38.5°, 48.8°, 53.5°, 58.06°, 61.48°, 66.15°, 68.1°, and 75.21° according to the JCPDS No. 48–1548, and various diffraction planes of (110), (002), (111), (20$\bar{2}$, (020), (202), (11$\bar{3}$), (113), (311), and (004) were presented. The XRD analysis of CuO and ZnO found no other impurities peaks [31,32]. XRD patterns of as-prepared nanocomposites of CuO/20% ZnO were obtained in the 2θ range of 10–90, as shown in Figure 3. The characterized peaks of CuO/20% ZnO demonstrate that the highly intense peaks are crystalline form. The CuO peaks recorded at 32.6, 35.8, 38.6, 48.5, 53.4, 58.3, 61.4, 75.4, and 83.1 correspond to the plane (110), (11$\bar{1}$), (111), (20$\bar{2}$), (020), (202), (11$\bar{3}$), (20$\bar{4}$), and (312), respectively. The observed pattern closely resembles the predetermined JCPDS pattern (JCPDS No. 41–0254). At the planes (200), (112), and (004), the ZnO peaks are found at 66.1, 68.0, and 72.1, respectively. The observed pattern closely resembles the standard JCPDS No. 36–1451. The presence of phase-pure hexagonal ZnO nanoparticles in the substance is guaranteed. The creation of a CuO/20% ZnO nanocomposite, consisting of monoclinic CuO with a space group of C2/c and hexagonal ZnO with a space group of P63mc, is shown by structural investigation. The exceptional quality and crystallinity of the powders are shown by the clearly defined experimental diffraction peaks for CuO/20% ZnO nanocomposite. According to Scherrer’s formula (Equation (2)), the hybrid CuO/20% ZnO nanocomposites were calculated as crystallite size (*D*) of the predominant peak of CuO 12.4 nm (11$\bar{1}$), 11 nm (111), and ZnO 14.5 nm (200) and 12.5 nm (112), respectively.

(2) $$D=\frac{k\lambda }{\beta cos\theta \;}\;\;$$

where k is constant, λ is wavelength, and *β* are full-width half-maximum values, respectively. The observed increase in crystallite size was driven by straining that occurred in the Cu^2+^ sites of the CuO host lattice as a result of Zn^2+^ inclusion. It is possible to explain the increase in crystallite size by the slight difference in Cu^2+^ (0.073 nm) and Zn^2+^ (0.074 nm) ionic radii. The remarkable crystallinity of the as-formed ZnO nanoparticles is demonstrated by the strong diffraction peaks, and it has exhibited as with cell constants of CuO corresponding to the a = 4.685 Å, b = 3.423 Å, c = 5.132 and ZnO belonging to the a = 3.25 Å, c = 5.21 Å, respectively. Furthermore, the XRD pattern’s broadening of the diffraction peaks indicates the existence of microstrain (ε) in the corresponding sample. Crystal defect and distortion have the potential to produce microstrain in the sample, and this can be computed using Equation (3). At the hkl planes of (11$\bar{1}$) and (111), the microstrain of CuO may be computed as 0.05 × 10^−3^ and 1.15 × 10^−3^, while the microstrain of ZnO was calculated as 0.302 × 10^−3^ and 0.203 × 10^−3^, corresponding to the (200) and (112) crystal planes, respectively. The structural parameter determined from the CuO/20% ZnO nanocomposite is presented in Table 1.

(3) $$\varepsilon =\frac{\beta }{4tan\theta }\;\;$$

**Figure 4:**
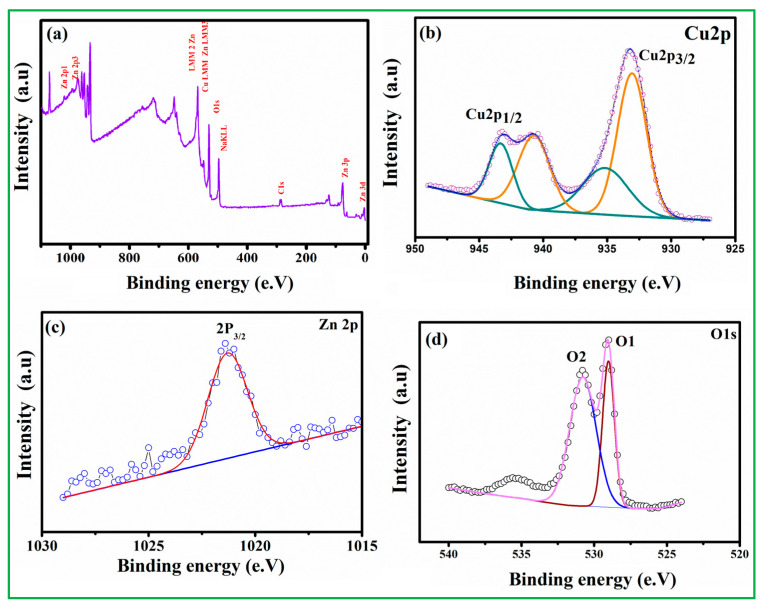
The XPS analysis of CuO/20% ZnO (**a**) survey spectrum, (**b**) Cu 2p, (**c**) Zn 2p, and (**d**) O 1sspectrum.

XPS tests were performed to investigate the elemental composition and valence state of the CuO nanoparticles. The survey spectrum is shown in Figure S2A, with peaks corresponding to the binding energies of atomic orbitals of Cu 2p and O 1s presented in CuO nanoparticles. The two peaks near 950.9 eV and 93.0 eV in the Cu 2p elemental spectra are Cu 2p_1/2_ and Cu 2p_3/2_, respectively (Figure S2B), and the signal splitting between these two states is about 20 eV [33,34,35]. Another O1S peak (Figure S2C) has three binding energies of 528.09 eV, 529.04 eV, and 529.73 eV, which belong to the middle, higher, and lower BE peak and then assigned to labile oxygen, oxygen vacancies, and chemically adsorbed oxygen (O^Chem^) on the surface. The absence of any other prominent peak in the XPS spectrum demonstrates that the prepared copper oxide is of great purity [36,37]. As seen in Figure 4a, the XPS survey scan spectra verify the existence of all relevant elemental peaks, including O 1s, Cu 2p, and Zn 2p. The two peaks, which are centered at 933.1 and 943.7 eV and correspond to Cu 2p_3/2_ and Cu 2p_1/2_, respectively, are shown in Figure 4b. Deconvolutions of the Cu 2p_3/2_ and Cu 2p_1/2_ yield two peaks at 933.2 eV (940.5 eV) and 935.5 eV (943.7 eV), respectively, corresponding to CuO (the Cu^2+^ oxidation state). The peak for Zn 2p3/2 is located at 1021.5 eV, as shown in Figure 4c, which represents the Zn 2p core level. The peaks of Zn 2p_3/2_ at 1021.5 eV are attributed to Zn–O– (hydroxide group bonding at the zinc surface) and ZnO (Zn^2+^ in the wurtzite ZnO structure), respectively. The presence of CuO and ZnO in the nanocomposite during the thermal reduction process via hydrothermal procedure is confirmed by the Cu 2p and Zn 2p spectra. Two peaks (O1 and O2) are identified in the binding energy of O 1s (Figure 4d). The first peak, located at 529.3 eV, corresponds to chemisorbed oxygen species (O^2−^) in the Zn–O bonding of the ZnO wurtzite structure, while the second peak, located at 530.6 eV, is related to oxygen-deficient regions (O^−^ and O^2−^ ions) in the sample matrix.

### 3.2. Electrochemical Characteristics of Electrodes

Electrochemical impedance spectroscopy (EIS) was used to evaluate the interface electron transfer resistance using a redox probe of 5.0 mM [Fe(CN)_6_]^3−/4−^ ferrocyanide/ferricyanide solution. The Nyquist diagram is composed of a high-frequency semicircle related to the electron transfer-limited process and a low-frequency linear part related to the diffusion-limited process [38]. It is obvious from the EIS diagram of equivalent circuit fit (Figure S5A,B) that the charge transfer resistance of CuO is very large (curve a), and the charge transfer resistance is 11.4 kΩ, which is much larger than ZnO and ZnO–CuO/GCE. The charge transfer resistance of zinc oxide is 144 Ω (curve b). It is determined that zinc oxide has a three-dimensional structure due to the flower shape, which can effectively provide charge distribution, thus improving electron transfer mechanics and providing the diffusion of ions to the electrode surface [39]. More importantly, after the ZnO–CuO composite modified GCE, the charge transfer is dramatically reduced to 131 Ω (curve c), which means that the ZnO composite CuO helps to reduce the resistance and thus increases the electrical conductivity.

### 3.3. Cyclic Voltammetry (CV) Test for Malathion Detection by Electrodes

**Figure 5:**
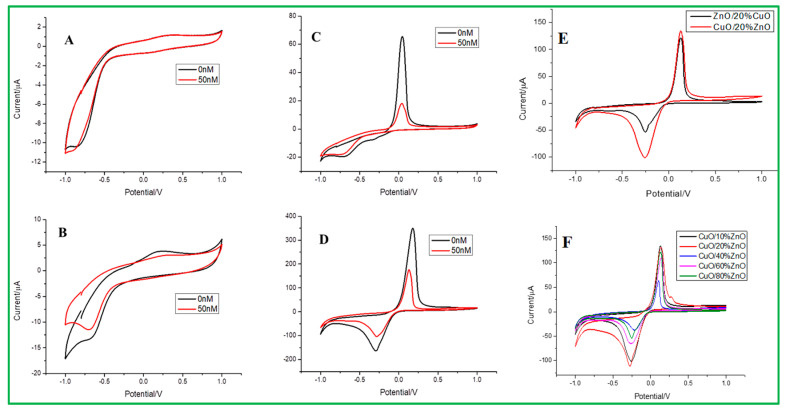
The CV data for (**A**) bare electrode, (**B**) ZnO/GCE, (**C**) CuO/GCE, (**D**) ZnO–CuO/GCE, in 0.2 mol/L Na_2_HPO_4_-0.1 mol/L citrate buffer solution (pH = 3.0) at the scan rate of 0.1 v/s. (**E**) Comparison study of ZnO/20% CuO, CuO/20% ZnO, and (**F**) CV data for different weight percentages of ZnO from 10 to 80%.

Electrochemical sensing of malathion at different electrodes was examined, as presented in Figure 5. The cyclic voltammetry (CV) data measurements were carried out at different electrodes, such as bare GCE, ZnO/GCE, CuO/GCE, and ZnO–CuO/GCE, in 0.2 mol/L Na_2_HPO_4_ with 0.1 mol/L citrate buffer solution (pH = 3.0). As shown in Figure 5A, B, the bare and ZnO-modified GCE did not show any redox peaks in the presence of 50 nM malathion and absence (0 nM malathion) of analytes. On the contrary, both CuO and ZnO–CuO/GCE can have obvious redox peaks in the presence and absence of analytes, as revealed in Figure 5C,D. In ZnO–CuO/GCE (Figure 5D), the peak position of the strong anode is +0.18 V (oxidation peak), and the cathode peak value is −0.3 V (reduction peak), which is compared to CuO/GCE (Figure 5C) that the upper strong anode peak is +0.04 V, and the cathode peak is more prominent at −0.96 V. In addition, the ZnO–CuO/GCE exhibited higher current density values than CuO/GCE in 0.2 mol/L Na_2_HPO_4_ with 0.1 mol/L citrate buffer solution (pH = 3.0). It can be explained that when 3D flower-like ZnO is used as a template to assist CuO nanoparticles, the electron transfer between CuO and the electrode can be enhanced, where the stronger redox peak value is exhibited. It can also be seen that when the concentration of malathion is higher, the peak will be suppressed due to the reaction of Cu(II)→Cu(0) change in the CuO valence state between CuO and malathion [40]. In addition, the detailed sensing mechanism of ZnO–CuO nanocomposites modified GCE for electrochemical detection of malathion is shown in Scheme 1. For comparison, it can be found that the current density of CuO/20% ZnO is higher than that of ZnO/20%CuO nanocomposites. Therefore, we first selected CuO/20% ZnO as the better condition, as revealed in Figure 5E. The different weight percentages of ZnO nanoparticles from 10 to 80% dispersed on the 3D-flower-like CuO are shown in the CV study (Figure 5F). According to these data, the CuO/10% ZnO catalyst exhibits better detection of malathion pesticides than other proportions of CuO/xZnO (X = 20, 40, 60, 80%).

### 3.4. Different Pulse Voltammetry (DPV) for Malathion Detection

**Figure 6:**
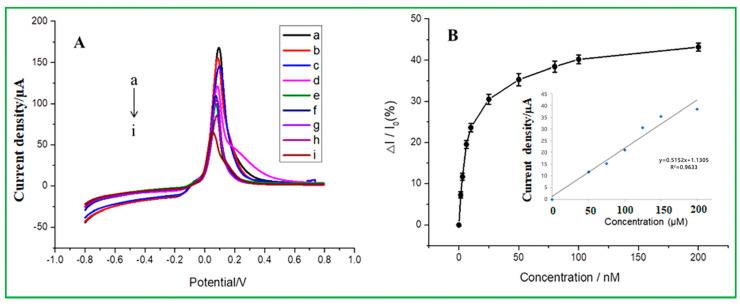
(**A**) DPV of ZnO–CuO/GCE at different concentrations of malathion in 0.2 mol/L Na_2_HPO_4_ with 0.1 mol/L citric acid buffer solution (from top to bottom, 0, 20, 30, 40, 50, 60, 80, 100, and 200 nM) (**B**) The relationship between the inhibition rate and different malathion concentrations (the inset is a linear correlation calibration curve).

The linear range and detection limit values of malathion pesticides were determined via the differential pulse voltammetry (DPV), as presented in Figure 6. DPV tests were carried out using the modified electrode of ZnO–CuO/GCE under the potential window of −0.8 V to 0.8 V in the buffer solution of 0.2 mol/L Na_2_HPO_4_ with 0.1 mol/L citric acid at pH = 3. As revealed in Figure 6A, the peak current decreased when the malathion concentration increased from a to i at the oxidation peak potential of −0.09 V. Because copper-oxide (CuO) nanoparticles have a high affinity for malathion, they exhibit excellent electrochemical behavior in a blank buffer system, but when malathion is added to the solution, CuO’s electrochemical behavior is inhibited, allowing for indirect detection of the amount of malathion and reducing their ability to transmit electrons to an electrode surface. The 3D nanoflower of ZnO boosts the conductivity of the electrodes and offers a larger surface area for the dispersion of CuO-NPs. The selective adsorption of malathion on CuO-NPs can obstruct the redox process on the surface of CuO-NPs, as the electrochemical behavior suggested. As shown in Figure 6B, the calibration curve shows that the inhibition ratio of malathion (Δ*I*/*I*_0_) gradually increases to a gentle level, indicating that malathion has reached adsorption saturation [41]. The inhibition ratio is directly proportional to the concentration of malathion, ranging from 0 to 200 nM. The linear equation is I_pa_ = 0.5152(C_malathion_) + 1.1305 and exhibits a correlation coefficient of R^2^ = 0.9633 and a detection limit of 1.367 nM. Table 2 summarizes and compares the detection limit of this investigation with previously reported literature for the detection of malathion. When compared to previous findings on the nonenzymatic electrochemical sensor, it exhibits better performance. Compared to previously reported electrochemical sensors, this nonenzymatic sensor, as prepared in this study, has a comparatively lower detection limit. Compared to the other references, it is found that the minimum detection limit obtained by ZnO–CuO/GCE in our study is lower than many reported electrochemical sensors. The minimum detection limit for AChE/Fe_3_O_4_ NPs CNTs modified electrode is 33.036 ppb (100 nM), or ZnO-AChE/GCE minimum detection limit is 35 ppb. According to the aforementioned discussion, it is expected that the catalyst CuO may create a sulfur-containing group with the sulfur-containing organic phosphorus molecules. Since CuO is well uniformly dispersed over the flower-like ZnO, it can result in a high oxidation peak value. Another point worth noting is that sulfur-containing groups are formed between malathion and CuO, and this chemical bond is too stable to allow ZnO–CuO/GCE to be regenerated. 

### 3.5. Optimization of the Experimental Conditions

#### 3.5.1. Effect of pH

**Figure 7:**
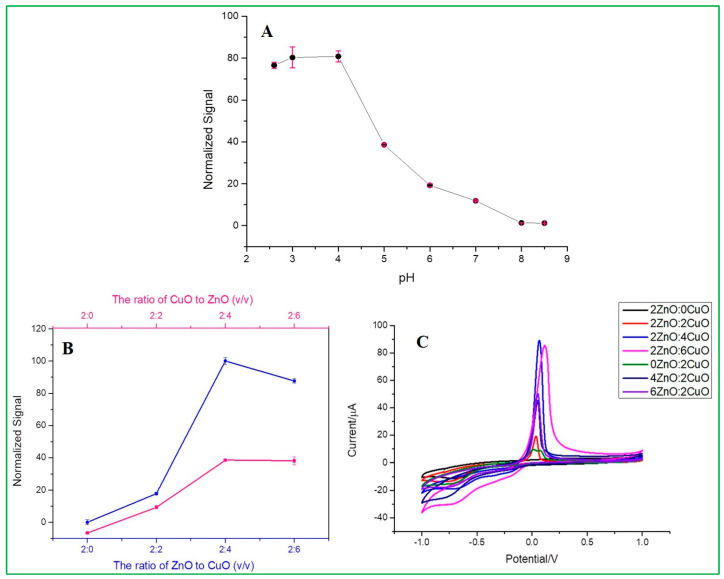
(**A**) Effects of pH, (**B**,**C**) Effect of volume ratio of as-prepared different weight proportion of ZnO and CuO nanocomposites.

The electrochemical signal diagram (Figure 7A) shows the effect of pH on the detection of 167 nM malathion by Nafion/ZnO-CuO/GCE prepared in 0.2 mol/L Na_2_HPO_4_ with 0.1 mol/L citric acid buffer solution, which can be clearly seen that the highest signal value is achieved at pH 3. Therefore, a buffer solution of pH = 3 was used for subsequent experiments. From the data analysis (Figure S3A), it can be seen that the current signal is almost consistent at pH = 3 and pH = 4, which represents that when the water sample is under an acidic environment, the as-prepared electrode can reach the highest signal value. On the contrary, the current signal would decrease under an alkaline environment. From the analysis results of zeta potential, as seen in Figure S3B, it can be clearly understood that the higher sensitivity in an acidic environment is because copper oxide has a relatively high and stable electronegativity at pH = 2, pH = 3, and pH = 4, compared with other pH values, and copper oxide is the catalyst that mainly forms redox peaks with malathion in the electrochemical analysis. Therefore, pH = 3 can be used as the optimal experimental parameter.

#### 3.5.2. Effect of Volume Ratio

**Figure 8:**
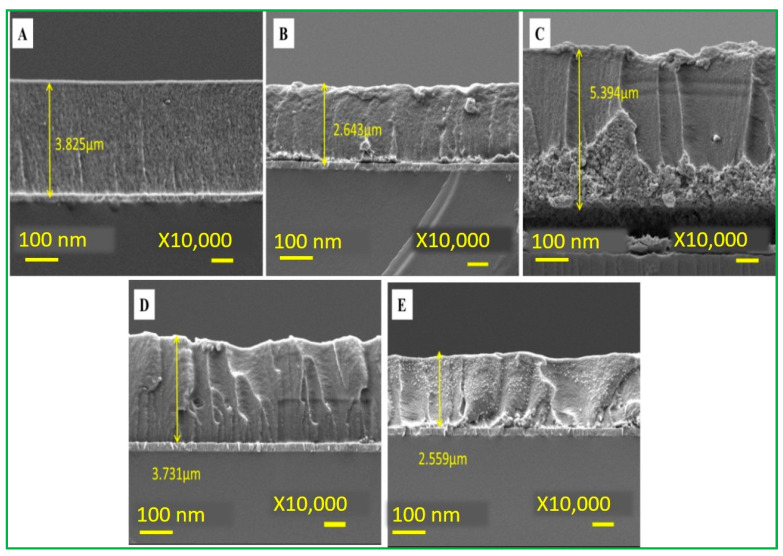
Electrode surface thickness (**A**) 2ZnO-2CuO (**B**) 2ZnO-4CuO (**C**) 2ZnO-6CuO (**D**) 4ZnO-2CuO (**E**) 6ZnO-2CuO.

This experiment was conducted under the above optimal pH condition. The CV data (Figure 7C) showed different current signals for different electrodes at a fixed scan rate of 100 mV/s. Among these, there is an optimal signal response obtained at a ratio of 2ZnO-4CuO (*v*/*v*), which was then selected for subsequent operation. This result also showed that the specific adsorption of malathion to copper oxide has inhibited the electrochemical peak value by the reduction of Cu(II) into Cu(0). When the ratio of copper oxide (CuO) is higher, the inhibition rate also increases, as revealed in Figure 7B. Furthermore, an increase in the ratio will cause a slight decrease in the inhibition, as seen in the electrochemical spectrum of the highest 2ZnO-4CuO (*v*/*v*) and the second-highest 2ZnO-6CuO (*v*/*v*). This result can be attributed to the increase in the film thickness, which suppresses the electron transfer and mass transfer processes of the modified electrode. The SEM data (Figure 8) show the film thickness at different ratios of ZnO–CuO modified electrode. The thickness of 2ZnO-4CuO (*v*/*v*) is 2.634 µm, which is the least compared to other ratios, except for 6ZnO-2CuO, which offers a better electrochemical performance towards the analysis of malathion. The electrode surface thickness calculated by SEM image is shown in Table S1.

#### 3.5.3. Effect of Different Response Time

**Figure 9:**
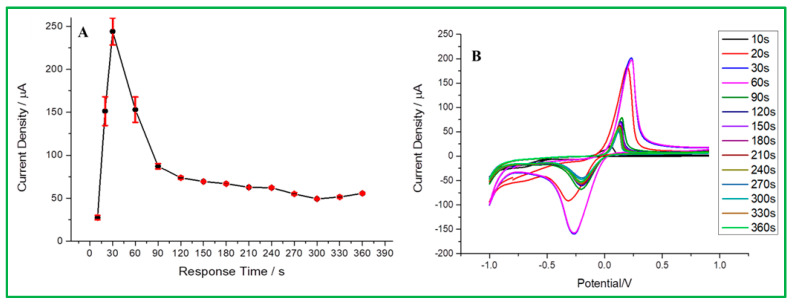
(**A**) Response time vs. current signal, (**B**) CV curve for different response times.

According to the above-mentioned best conditions for this experiment, the detection of malathion is based on the anode surface adsorption, so the response time before analysis will affect the signal intensity in malathion analysis. According to Figure 9A, it is obvious that the optimal response time is 30 s. The test was started and found to have the highest current density until 30 s. However, the current response tended to significantly decline with a longer response time because malathion has reached adsorption saturation on the electrode surface. In the CV data (Figure 9B), the current signal at 30 s is about 201 µA, the signal height at 20 s is 184.95 µA, and 60 s is 196.79 µA. Therefore, 30 s was determined to be the optimal response time to maintain sensitivity and efficiency for malathion analysis.

### 3.6. Selectivity Test

**Figure 10:**
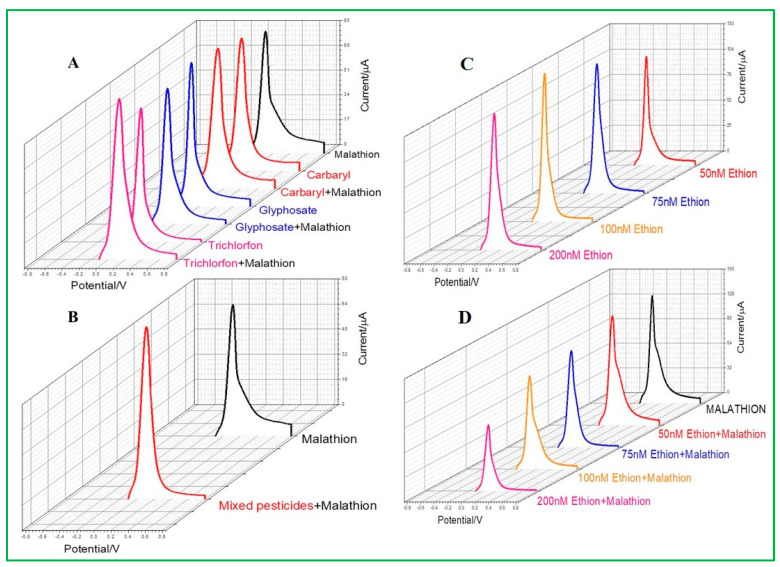
(**A**) Selectivity test of combined addition of different pesticides such as carbaryl glyphosate and trichlorfon pesticides and mixed malathion, (**B**) mixed pesticides and added malathion, (**C**) DPV of ethion, and (**D**) different concentrations of ethion with malathion.

The study of pesticide selectivity becomes crucial since a range of pesticides, including insecticides and herbicides, are frequently utilized in agricultural operations. In this study, four types of pesticides, such as ethion, glyphosate, trichlorfon, and carbaryl, were chosen with their similar chemical properties as malathion. Among these, ethion is the only sulfur-containing organophosphorus pesticide. It was observed from differential pulse voltammetry that neither carbaryl, glyphosate, nor trichlorfon would selectively interfere with the malathion detection by ZnO–CuO/GCE (Figure 10A). All current signals are similar in height compared to malathion alone because the detection is based on the interaction between copper and sulfur groups. If the same concentration of pesticides of carbaryl, glyphosate, trichlorfon, and malathion are present in the same water sample at the same time, the results show that ZnO–CuO/GCE will not be interfered with by other pesticides (Figure 10B), indicating that the as-prepared electrode is highly selective. Another sulfur-containing pesticide, ethion, was selected for testing, as revealed in Figure 10C. It was observed that the detection of ethion would also produce a current value, which means that ZnO–CuO/GCE can be interfered with by the same sulfur ions containing malathion. The current height of malathion was not affected by ethion until its concentration added up to 200 nM, despite the fact that two sulfur-containing pesticides were mixed and evaluated simultaneously, as demonstrated in Figure 10D. Therefore, it can be concluded that ZnO–CuO/GCE is selective for malathion analysis if the moderate concentration of co-existing sulfur-containing pesticides.

### 3.7. Reproducibility and Stability Test

**Figure 11:**
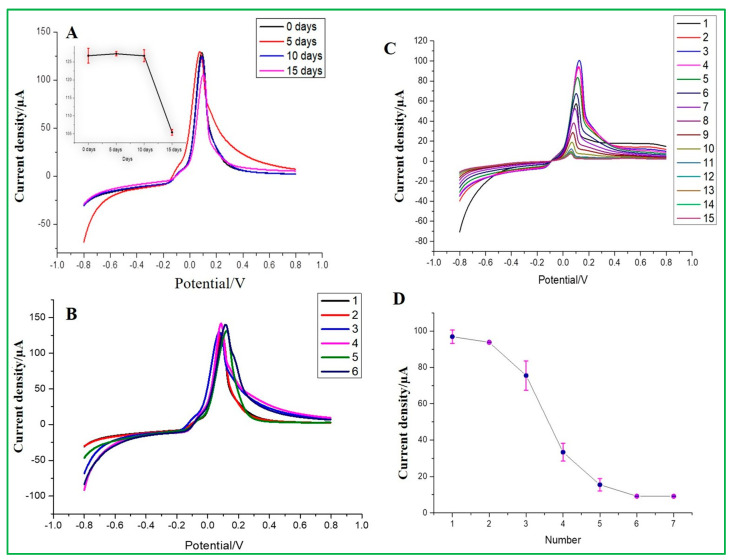
(**A**) Stability of storage days in a room environment (inset current vs. days), (**B**) Stability of sensing electrode, (**C**) modified electrode repeated detection of malathion 15 times, and (**D**) current density vs. the number of times used.

The reproducibility of ZnO–CuO/GCE was examined by changing different stored times for 0, 5, 10, and 15 days in a room environment and repeatedly detecting the same concentration of 167 nM malathion. It can be seen from the electrochemical results that at the stored time of 15 days, the current signal would drop significantly, where the inset data are presented in Figure 11A. From the results, it can be estimated that the modified ZnO–CuO/GCE electrode can be stored in a dry environment for approximately 10 days. Meanwhile, the entire process was repeated six times to observe its stability when the same electrode was repeatedly used and washed. As shown in Figure 11B, it can be seen that the peak height of the electrochemical signal is basically at the same position, but the peak current started to become lower due to the inhibition of electron transfer between pesticides and modified electrode materials. In the meantime, the results exhibited that the RSD value of current values measured by 6 modified electrodes was 3.2%, indicating that the sensor had good reproducibility. Finally, the repeatability test was carried out using as-prepared ZnO–CuO/GCE and used for 15 repeated detections of malathion at 167 nM concentration under the best conditions. As a result, it can be seen from the results in Figure 11C,D that the first and second test results have the same current signal. After the third test, the current height began to decline. This result exhibits that the number of detections per electrode can be used for precisive measurement twice, but the data after two detections will gradually lose its detection accuracy. Although there was a little change in the morphology as revealed by the ZnO–CuO/GCE post-characterization analysis (Figure S4), the atomic and weight percentage of element analysis was validated by the EDX spectrum to be in nearly good agreement between the pre-and post-15-day stability cycles.

## 4. Conclusions

In conclusion, a new nonenzymatic electrochemical sensor based on the ZnO–CuO/GCE modified electrode was synthesized for the detection of malathion in aquatic environments. According to experimental findings, the CuO nanoparticles in nanocomposites were shown to have a strong affinity for malathion, whereas the presence of ZnO shown in 3D-nanoflower will improve the ability of CuO to transfer electrons to the electrode and enhance the ability to detect malathion electrochemically. Furthermore, the DPV results showed that the malathion linear range from 0 to 200 nM, and the detection limit is 1.367 nM. According to experimental findings, this electrochemical sensor can detect organophosphorus pesticides that include sulfur groups and has good stability and reproducibility. These findings have demonstrated that the designed ZnO–CuO/GCE nanocomposites sensor is an appealing candidate for fast, easy, and sensitive analysis of malathion in an aqueous environment. For the analysis of malathion detection by ZnO–CuO/GCE modified electrode, the suggested nonenzymatic methodology has proved to be easy to use, effective, and economical. It is a viable alternative that may be explored in the future. The harmful effects of various organophosphate pesticides on human health can also be detected using the new electrochemical enzyme-free sensing devices. Future improvements should be made to the feasibility study on the manufacturing of nonenzymatic electrochemical sensors. In addition, we should optimize the detection limits with improved selectivity of various OP pesticides detection by the modified electrode of ZnO–CuO/GCE. 

## Figures and Tables

**Table 1:** The structural parameter calculated from the XRD patterns of CuO/20% ZnO nanocomposite.

Electrode	Crystalline Size	Lattice Parameter	Macrostrain
CuO	12.4 nm (11$\bar{1}$) 11 nm (111)	a = 4.685 A° b = 3.423 A° c = 5.132 A°	0.05 × 10^−3^ (11$\bar{1}$) 1.15 × 10^−3^ (111)
ZnO	14.5 nm (200) 12.5 nm (112)	a = 3.25 A° c = 5.21 A°	0.302 × 10^−3^ (200) 0.203 × 10^−3^ (112)

**Table 2:** The comparative study of different types of chemically modified electrodes for the detection of malathion.

Electrode	Linear Range	Method	LOD	References
PANI-ZnO−NiO/GCE	0.01–0.07 µM	DPV	0.01 µM	[42]
PANI-ES/SWCNTs	0.2–1.4 µM	DPV	0.2 µM	[43]
c-MWCNT-CuO	20–300 nM	CV	0.143 nM	[44]
PA6/PPy/CRGO/FTO	1.7–67 µM	DPV	12.7 × 10^−3^ µM	[45]
BTCA-P-CuCP/CPE	0.6 × 10^−3^–24.0 × 10^−3^	CV	0.2 × 10^−3^	[46]
CuO NWs–SWCNTs/GCE	0.3–1.4 nM	DPV	0.3 nM	[47]
Gly-CuO/GCE/Nafion	1–12 nM	DPV	0.1 nM	[48]
ZnO–CuO/GCE	0 to 200 nM	DPV	1.367 nM	This work

## Data Availability

Data can be available upon request from the authors.

## Supplementary Materials

The following supporting information can be downloaded at https://www.mdpi.com/article/10.3390/ma16227065/s1, Figure S1: (A) The optimization study of the modified electrode of ZnO–CuO/GCE at different pH and (B) Zeta potential values for different electrode materials, Figure S2: The XPS analysis of pristine CuO (A) survey spectrum, (B) Cu 2p, and (C) O 1S spectrum, Figure S3: (A) The optimization study of the modified electrode of ZnO–CuO/GCE at different pH and (B) Zeta potential values for different electrode materials, Figure S4: (a,b) TEM and EDX spectrum of ZnO–CuO/GCE spectrum for post-analysis following 15 stability tests, Figure S5: (A) EIS spectrum in a 0.1 M KCl solution containing 5.0 mM [Fe(CN)_6_]^3−/4−^ electrode Nyquist graphs of (a) CuO/GCE, (b) ZnO/GCE, (c) ZnO–CuO/GCE and (B) the equivalent circuit fit of the pristine CuO, ZnO, and 3D-ZnO–CuO nanocomposites, and Table S1: The electrode surface thickness calculated by SEM image.
