# Supplementary material for: Detection of Environmentally Harmful Malathion Pesticides Using a Bimetallic Oxide of CuO Nanoparticles Dispersed over a 3D ZnO Nanoflower
Source: Materials (Basel). 2023 Nov 7;16(22):7065. doi: 10.3390/ma16227065 (PMC10672217; doi:10.3390/ma16227065)
Supplement: Supplementary file 1 [file materials-16-07065-s001.zip › materials-2644225-supplementary.pdf]

Supporting Information

Detection of Environmentally Harmful Malathion Pesticides Using Bimetallic Oxide of CuO Nanoparticles Dispersed over a 3D-Nanoflower ZnO

Lakshmanan Gurusamy[1], *Ru-Wen Cheng*[1], *Sambandam Anandan*[2], *Cheng-Hua Liu*[1], *and Jerry J. Wu*[1,*]

[1] *Department of Environmental Engineering and Science, Feng Chia University, Taichung 407, Taiwan*

[2] *Department of Chemistry, National Institute of Technology, Trichy, India*

* Corresponding author: jjwu@mail.fcu.edu.tw

1. Preparation of zinc oxide

Flower-like ZnO hollow microspheres were synthesized by a simple hydrothermal method. The ZnO microspheres were synthesized according to other literature methods . All reagents are analytical grade and can be used directly without further purification.

First, 0.439 g of $Zn(CH_3COO)_2{\cdot}2H_2O$, 0.4 g of glycine, and 0.4 g of Na_2SO_4 $10H_2O$ were completely dissolved in 15 mL of deionized water and 10 ml of ethanol while vigorously stirring for 5 minutes. After five minutes, 0.4 g of sodium hydroxide was added to the above solution and stirred for about 1 hour. Next, the mixed solution was transferred to a Teflon stainless steel autoclave, and heated at 180°C for 12 hours. After cooling, the precipitate was washed and collected in distilled water and alcohol by centrifugation, and repeated several times. Finally, put it in an oven at 70°C for 12 hours, and put it in an autoclave at 400°C for 2 hours.

2. Preparation of copper oxide

Copper oxide was synthesized by hydrothermal method. The copper oxide was synthesized by referring to other literatures. All chemicals are analytical grade and can be used without further purification.

The production process is shown in Figure 1. First, copper nitrate is used as the copper precursor, and the best conditions are found from 0.025 M, 0.05 M, 0.1 M, 0.15 M, and 0.2 M. Next, adjust the concentration of sodium hydroxide to find the best parameters from 0.5 M, 1 M, 1.5 M, 2 M, and 2.5 M. Finally, the first two parameters are integrated, and the copper precursors are changed to copper sulfate, copper nitrate, copper acetate and copper chloride to find the best conditions.

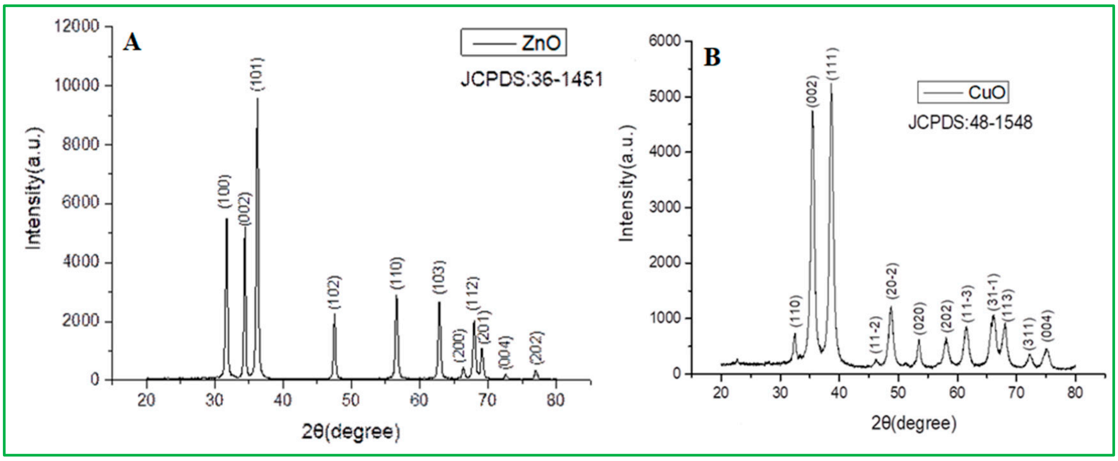

Figure S1. The XRD patterns of pristine (A) ZnO and (B) CuO

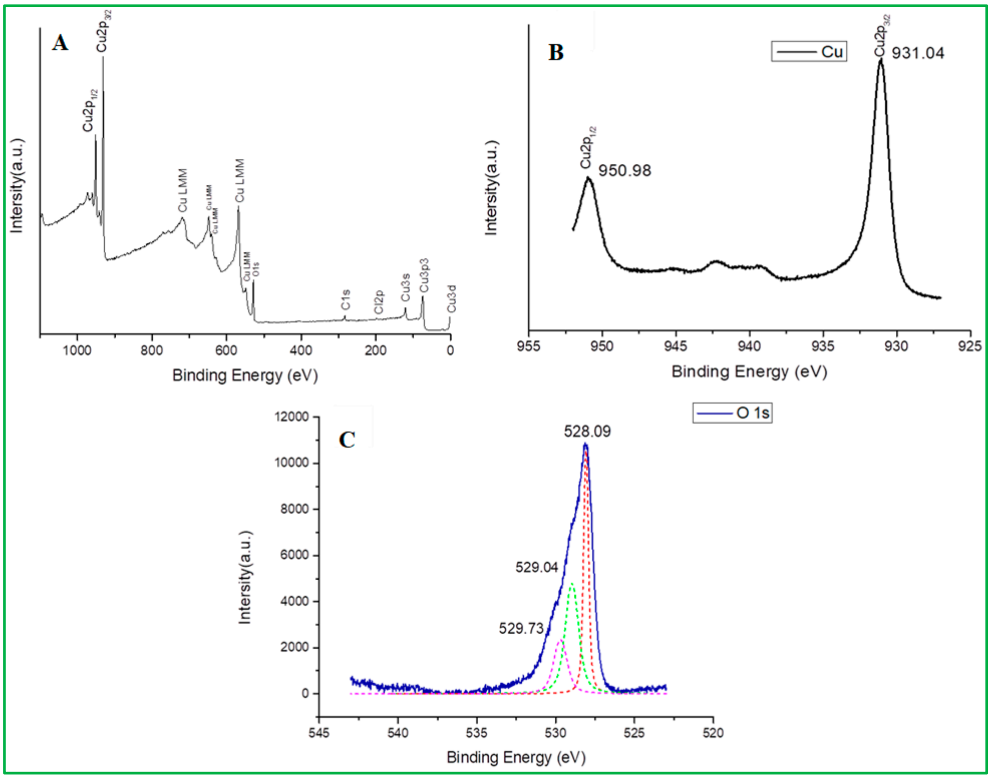

Figure S2. The XPS analysis of pristine CuO (A) survey spectrum, (B) Cu 2p, and (C) O 1S spectrum

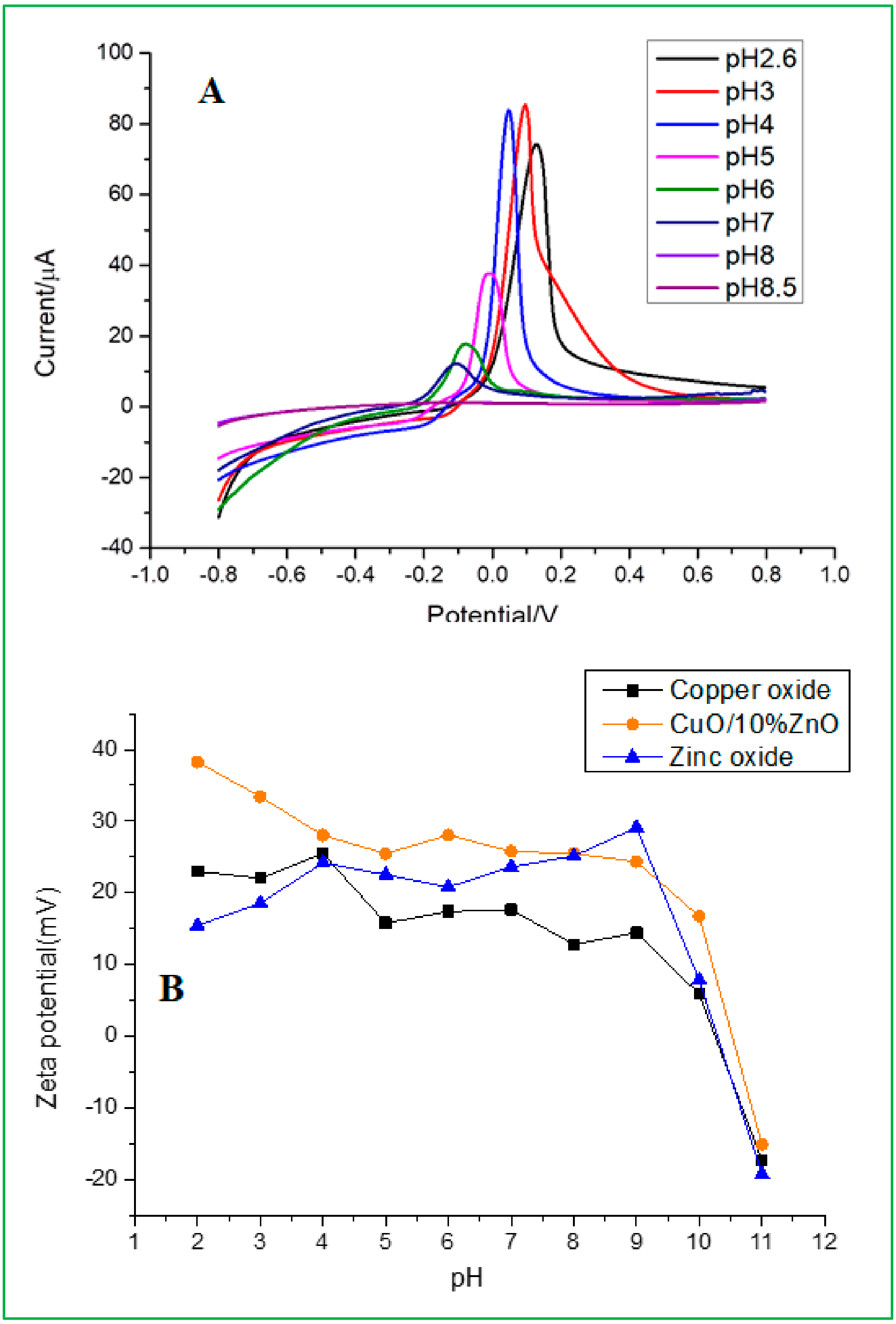

Figure S3. (A) The optimization study of the modified electrode of ZnO-CuO/GCE at different pH and (B) Zeta potential values for different electrode materials

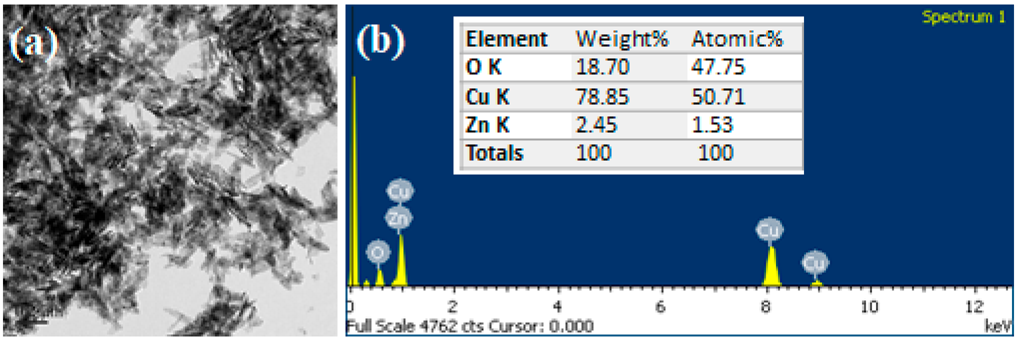

Figure S4. (a,b) TEM and EDX spectrum of ZnO-CuO/GCE spectrum for post-analysis following 15 stability tests

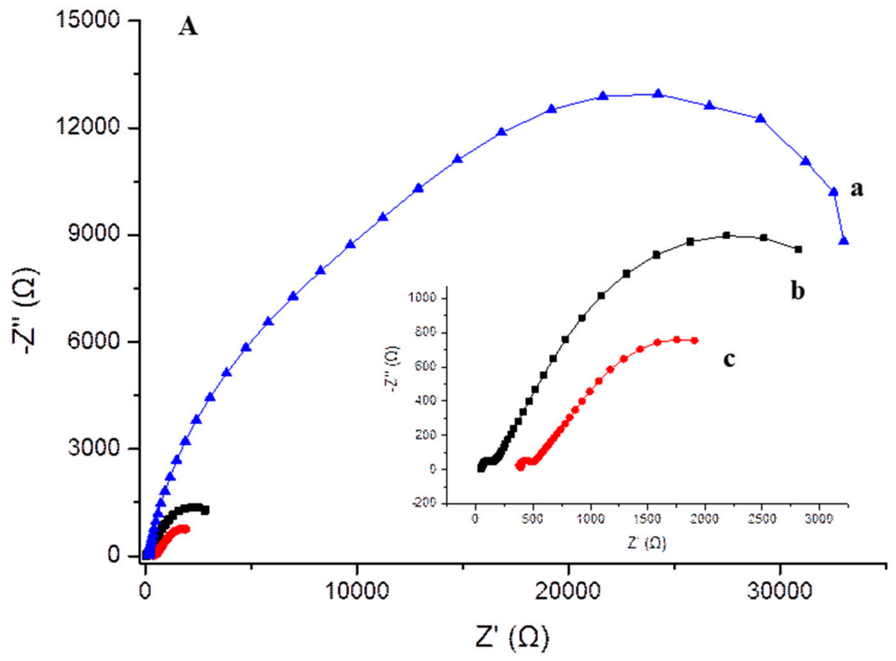

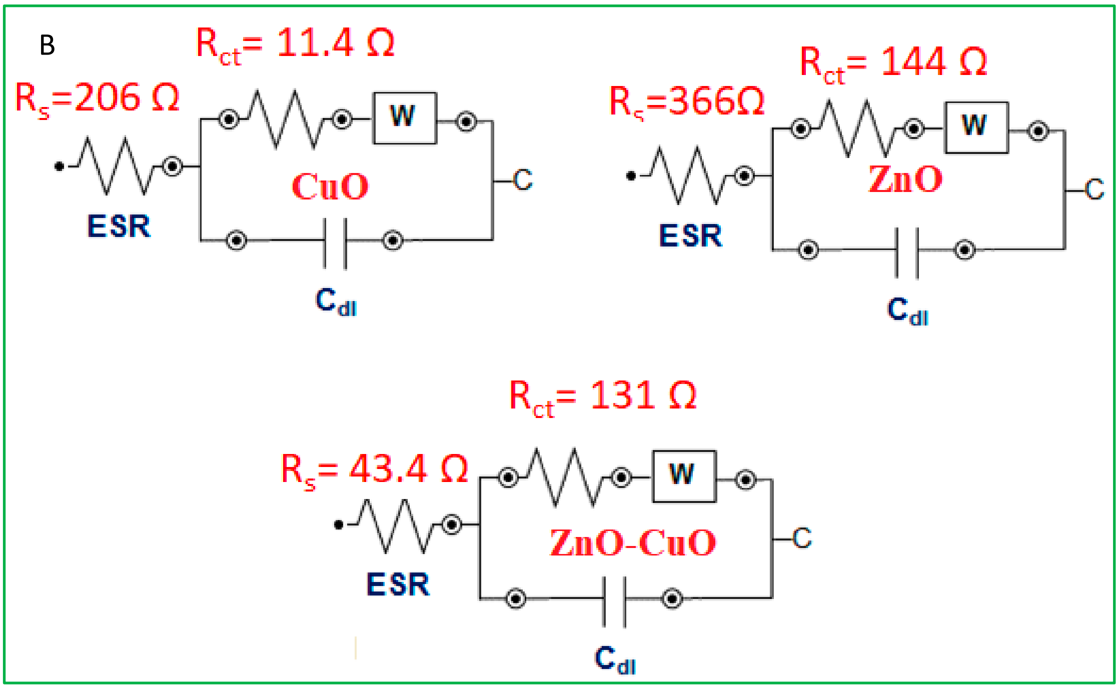

Figure S5. (A) EIS spectrum in a 0.1 M KCl solution containing 5.0 mM $[Fe(CN)_6]^{3-/4-}$ electrode Nyquist graphs of (a) CuO/GCE, (b) ZnO/GCE, (c) ZnO-CuO/GCE, and (B) The equivalent circuit fit of the pristine CuO, ZnO, and 3D-ZnO-CuO nanocomposites

Table S1. The electrode surface thickness calculated by SEM image

Electrode	surface thickness
2ZnO-2CuO	3.285 µm
2ZnO-4CuO	2.643 µm
2ZnO-6CuO	5.394 µm
4ZnO-2CuO	3.731 µm
6ZnO-2CuO	2.559 µm
